# Investigation of High-Temperature Oxidation Behavior of Additive Manufactured CoCrMo Alloy for Mandrel Manufacturing

**DOI:** 10.3390/ma17153660

**Published:** 2024-07-24

**Authors:** Adina Cristina Toma, Mihaela Raluca Condruz, Alexandru Paraschiv, Teodor Adrian Badea, Delia Pătroi, Nicoleta Mirela Popa

**Affiliations:** 1 Romanian Research and Development Institute for Gas Turbines COMOTI, 220D Iuliu Maniu Av., 061126 Bucharest, Romania; adina.toma@comoti.ro (A.C.T.); alexandru.paraschiv@comoti.ro (A.P.); teodor.badea@comoti.ro (T.A.B.); 2 National Institute for Research and Development in Electrical Engineering, ICPE-CA, 313 Splaiul Unirii, 030138 Bucharest, Romania; delia.patroi@icpe-ca.ro; 3 National Institute of Research and Development in Mechatronics and Measurement Technique—INCDMTM, Pantelimon Str. No. 6–8, 022401 Bucharest, Romania; popa_nicoleta2093@yahoo.com; 4 Doctoral School of Materials Science and Engineering, National University of Science and Technology Politehnica Bucharest, 313 Splaiul Independenței, 060042 Bucharest, Romania

**Keywords:** oxidation, CoCrMo, oxide scale, microstructural investigation, CoCr_2_O_4_, Cr_2_O_3_

## Abstract

The cyclic oxidation behavior of an additive manufactured CoCrMo alloy with 0.14 wt.% C was investigated at 914 °C for 32 cycles, each lasting 10 h, resulting in a total exposure time of 320 h. The oxidation rate was assessed for mass gain after finishing each 40 h oxidation cycle. It was experimentally determined that the oxidative process at 914 °C of this CoCrMo alloy follows a parabolic law, with the process being fast at the beginning and slowing down after the first 40 h. The microstructural analysis revealed that in the as-printed state, the phases developed were primarily the γ matrix and minor traces of ε phase. The oxidative process ensured an increase in the ε phase and precipitation of carbides which produced a 12% increase in the material’s hardness after the first 40 h of exposure at 914 °C. The oxidation process led to the development of an oxide scale comprising a dense Cr_2_O_3_ layer and a porous layer of CoCr_2_O_4_ spinel, the latter spalling after the 240 h of exposure. Despite this spallation, the oxide scale continued to develop in the presence of O, Cr, and Co. The experimental analysis provided valuable insights regarding the material’s behavior under prolonged exposure at high temperature in air, demonstrating its suitability as a candidate for additive manufactured mandrels used for bending metallic pipe fitting elbows.

## 1. Introduction

Additive manufacturing (AM) is a disruptive manufacturing technology of interest for both scientific and industrial communities. Current research efforts are concentrated on several key areas, such as the fabrication of intricate geometries and customized products [1,2,3], the development of novel materials compatible with various AM techniques [4], and the establishment of standardized protocols for AM processes. Among the AM methods, an increased interest was registered for metal AM, especially for the powder-based fusion method (PBF), including selective laser melting (SLM), selective laser sintering (SLS), and electron beam melting (EBM), but also for other methods that use metallic material feedstock [5].

AM is a technology used in various industries (e.g., medicine, aerospace), and numerous studies have been published regarding the AM of high-performance components, specially manufactured from Ni-based superalloys, with studies also focusing on the characterization of materials and the enhancement of their properties through various methods, including the optimization of process parameters [6,7,8,9,10,11,12]. Except for Ni-based superalloys, other alloys have become increasingly important for various high-performance industries, including CoCr alloys. These alloys are known for their excellent mechanical properties at room and high temperatures, including high wear, oxidation and corrosion resistance, high strength, and biocompatibility. These characteristics make them suitable for demanding applications, such as medical implants and prosthetics, cardiovascular devices, components for gas turbines’ critical components, or mandrels used to bend tubes and pipes for the aerospace and automotive industries [13,14,15,16,17].

The CoCr alloys date back to the 1920s when Elwood Haynes introduced the “Stellite” alloys. In the 1930s and 1940s, the excellent biocompatibility and corrosion resistance of CoCr alloys were recognized, leading to their use in dental prosthetics and in orthopedic implants. By the 1950s, CoCr alloys were introduced in the aerospace industry for high-temperature applications [18]. Regarding the composition of CoCr alloys, they consist of Co as the primary matrix material; it has a face-centered cubic (FCC) crystal structure that, when slowly cooled, transforms into a hexagonal close-packed (HCP) structure at temperatures around 450 °C. Cr is the main alloying element that ensures the mechanical strength and oxidation and corrosion resistance and acts as a carbide former (M_7_C_3_, M_23_C_6_). Except Cr, W and Mo can be added to enhance the matrix’s strength, the precise composition can vary depending on the desired application and performance requirements [18]. 

The advancement in material science and manufacturing technologies has made possible the use of CoCr alloys as suitable powder feedstock for AM. Various studies have been conducted regarding the microstructural tailoring of additive manufactured CoCr alloys to improve the material’s performances [19,20,21,22], the studies being conducted mainly in the biomedical field [23,24,25]. 

Even if studies were found regarding the characterization of additive manufactured CoCr alloys for biomedical field, in terms of alloys’ performance at high temperatures, the studies primarily target conventional manufactured materials. For example, Ayu et al. [26] studied the thermal oxidation response of a CoCrMo alloy heated for 3 h/6 h at 850 °C, and their study revealed that the corrosion resistance of a CoCrMo alloy can be increased by applying a thermal oxidation process that promotes an oxide layer formation on its surface. In a study performed by Comakli [27], it was discovered that the CoCrMo alloy’s oxidation at 850 °C for 3 h/5 h increased its wear resistance. Etienne et al. [28] studied the microstructure and oxidation behavior of CoCrTa alloys after oxidation in air at 1200 °C for 24 h, and they concluded that the materials exhibited a good oxidation resistance due to the formation of CrTaO_4_. Karaali et al. [28] studied the effect of W additions in CoCr alloys on their oxidation resistance in the range of 950 °C–1050 °C. They reported that the W addition does not change the morphology of the oxide layers formed, but reduces its thickness and slows the oxidation process, as it accumulates within the inner layer of the material, locking the Co diffusion towards the alloy’s surface.

The present study was focused on studying the additively manufactured CoCrMo alloy behavior after prolonged high-temperature exposure in air. The material was studied as a suitable candidate to AM various mandrels’ geometries for bending metallic pipes fitting elbows, but in a more cost-effective way compared to the conventional manufacturing technologies currently applied. Regarding the costs incurred in the conventional manufacturing of mandrels from CoCr alloys, it is known that the baseline materials used in the production of CoCr alloys are considered relatively expensive compared to other materials but are more stable. The casting of CoCr alloys is also an expensive process as they are developed in vacuum furnaces. By using AM, the material losses are reduced, the metallic molds used for casting are eliminated, and the mandrel’s weight can be reduced by integrating complex lattices inside without affecting its structural integrity. Furthermore, the AM process can ensure the production of mandrels that do not need mechanical finishing and periodic dimensional corrections.

## 2. Experimental Procedure

The experimental procedure included the manufacturing of specimens, performing the cyclic high-temperature oxidation treatment, and assessment of the influence of the oxidation cycles on the material’s features. Cylindrical rods of size 16 mm × 65 mm were additively manufactured using the machine EP-M250 PRO Dual-laser 3D printer from Shining 3D Tech Co., Ltd., Hangzhou, China and EOS CoCr MP1 powder purchased from EOS GmbH, Krailling, Germania. The process parameters used for rod manufacturing were as follows: temperature of the building plate 60 °C, chamber pressure 50 mbar, layer thickness 30 μm, laser parameters in terms of outer skin speed 400 mm/s and power 110 W, inner skin speed 400 mm/s and power 110 W, middle speed 893 mm/s and middle power 120 W, upskin speed 893 mm/s upskin power 120 W, downskin speed 600 mm/s, downskin power 140 W, hatch type stripe with field width 5 mm and field overlap 0.08 mm, and skinfill angle 30°. The scanning strategy (skin and core building style) was selected to increase the building speed. The manufacturing process took place in a nitrogen atmosphere using a stainless steel building plate.

The CoCr MP1 powder (powder from the UNS R31538 CoCrMo superalloy) is a material suitable for applications where high mechanical properties, corrosion resistance, and high-temperature performances are essential. It has a powder size distribution in the range of 16–51 μm. The material’s chemical composition is presented in Table 1.

All rods were mechanically detached from the building plate, and the supports were cut by wire electrical discharge machining. Specimens 12 mm high were cut from the rods, and a number of 18 specimens were used for the present study. The ends of the specimens were metallographically prepared by grinding on sandpaper and wet polishing with 3 µm diamond paste. No other chemical or mechanical preparation was performed for the specimens as the mandrel is intended to be used in the as-printed state without surface finishing. From the 18 specimens, 2 were kept as references, and the 16 others, along with Al_2_O_3_ crucibles, were used for the oxidation process. All 16 specimens and the crucibles were cleaned with ethanol and were weighted, with and without the crucibles, using the analytic balance Pioneer PX 224 (Ohaus Europe GmbH, Nänikon, Switzerland).

The high-temperature cyclic oxidation influence on the material’s behavior was assessed by performing isothermal cyclic oxidation heat treatment at 914 °C, in air, using the Nabertherm LH 30/14 furnace (Nabertherm GmbH, Lilienthal, Germany). The oxidation heat treatment consisted of heating from room temperature for 1.5 h until the temperature of 914 °C was reached, followed by maintaining for 10 h at temperature and cooling until room temperature with the furnace door open. A total of 32 stages of oxidation were performed until a total 320 h exposure time at 914 °C was reached. The heat treatment conditions were established based on the mandrel’s working conditions and its lifetime. A mandrel used for metallic piping forming has a lifetime of approximately 1000 working hours at a temperature in the range of 800–870 °C. To reduce the experiment’s duration by approximatively 70% (reaching 320 h of exposure time), the temperature was increased to 5% from the 870 °C, obtaining a value of 914 °C. No mechanical loads were taken into consideration during the present study. 

Investigations were performed on specimens after each 40 h cycle of exposure, 2 specimens being retracted from the batch. The specimens’ mass gain was assessed after finishing each 40 h oxidation cycle by weighing all the crucibles with the specimens inside. The microstructural analysis was performed by optic microscopy using the Axio Vert.A1 MAT (Carl Zeiss Microscopy GmbH, Jena, Germany) metallographic microscope with camera and dedicated analysis software NIS-Elements (Nikon Instruments Inc., Melville, NY, USA), v5.0, and scanning electron microscopy using FEI F50 Inspect SEM (FEI Company, Brno, Czech Republic) equipped with an energy-dispersive X-ray spectrometer (EDS) EDAX APEX 2i with SDD Apollo X detector. By SEM analysis, the morphology of the oxide layer was studied, as well as its thickness and chemical composition. To assess the microstructural changes in the material after high-temperature exposure, the specimens were cut in half, and one half was used for optical microscopy investigations and the other one for SEM analysis. The oxide scale thickness was determined by measurements performed in the cross-section on resin-mounted specimens by processing SEM images using the Scandium software (v5.2, Olympus Soft Imaging Solutions GmbH, Münster, Germany). Qualitative micro-compositional analysis was performed by SEM-EDS on the specimen’s cross-section, in various micro-areas located in the scale and base material region (substrate). The nominal chemical composition was determined by SEM-EDS also for the base material on reference specimens. The optical microscopy was performed in XY and XZ planes, on metallographically prepared surfaces by grinding and wet polishing. Further, they were etched with Acetic Glyceregia reactive agent (consisting in 15 mL HCl, 10 mL CH_3_COOH, 5 mL HNO_3_, 2 drops of C_3_H_8_O_3_) for microstructural feature highlighting and the assessment of microstructural evolution during multiple high-temperature oxidation cycles. 

The oxide scale phases’ nature was investigated by X-ray diffraction (XRD) using the D8 Discover diffractometer (Bruker, Bremen, Germany), with Cu primary radiation (λ = 1.540598Å), Goebel mirror and 1D LynxEye detector on the secondary side. The diffractograms were recorded with an angular increment of 0.04°, at a scanning speed of 1 s/step. The qualitative analysis was carried out using the PDF2 ICDD database.

The evolution of the material’s hardness was also analyzed over all the exposure period. The Rockwell hardness (HRC) was measured using the EMCO Test universal hardness measuring apparatus.

## 3. Results

**Figure 1:**
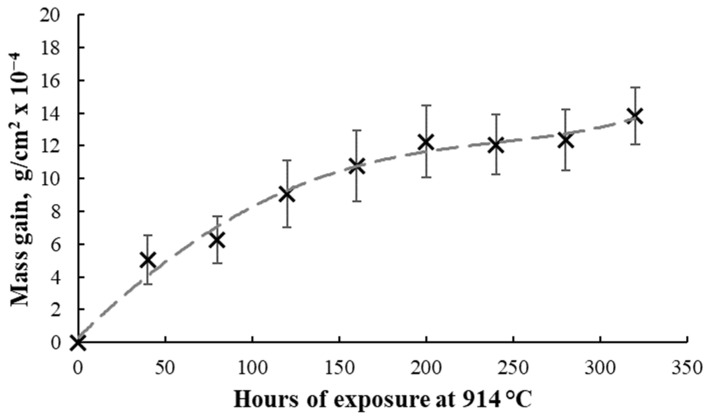
Kinetic curve of CoCr oxidation at 914 °C for 320 h of maintaining.

Figure 1 presents the kinetic curve of the isothermal oxidation of CoCrMo specimens for 320 h of exposure at 914 °C. The mass gain analysis results represent the difference between specimens’ weight after exposure and the initial weight relative to the specimens’ surface.

**Figure 2:**
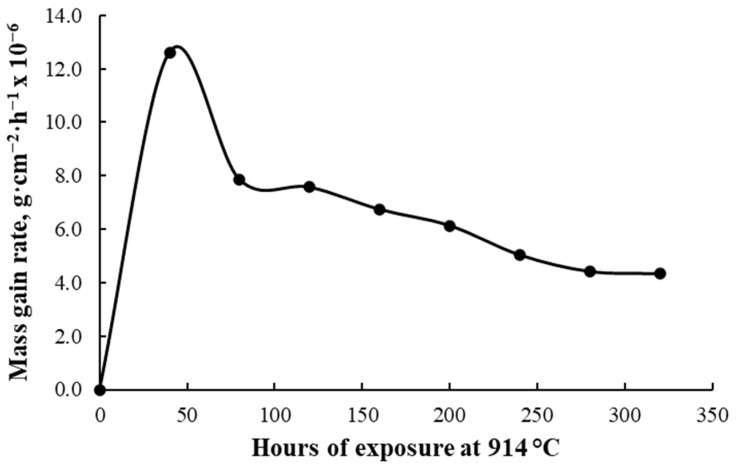
The mass gain rate of the oxidized specimens from additively manufactured CoCrMo as a function of time.

The graphical representation from Figure 1 reveals that the oxidation process of additively manufactured CoCrMo alloy follows a parabolic law, while the graphic representation from Figure 2 shows the mass gain rate calculated with the average mass gain of the oxidized specimens as a function of time. 

A significant increase in the mass gain during the first 40 h of exposure at 914 °C was observed, continuing with a constant increase until 200 h of exposure, and afterwards for two stages, the mass gain was kept relatively constant and further increased at the 320 h stage of exposure. This behavior shown after the 200 h exposure time can be caused by an oxide scale spallation due to thermal stresses or mechanical stresses in the oxide layer. The kp oxidation constant of the additively manufactured CoCrMo calculated for the 320 h of exposure was 16.7 × 10^−13^ g^2^·cm^−4^·s^−1^.

**Figure 3:**
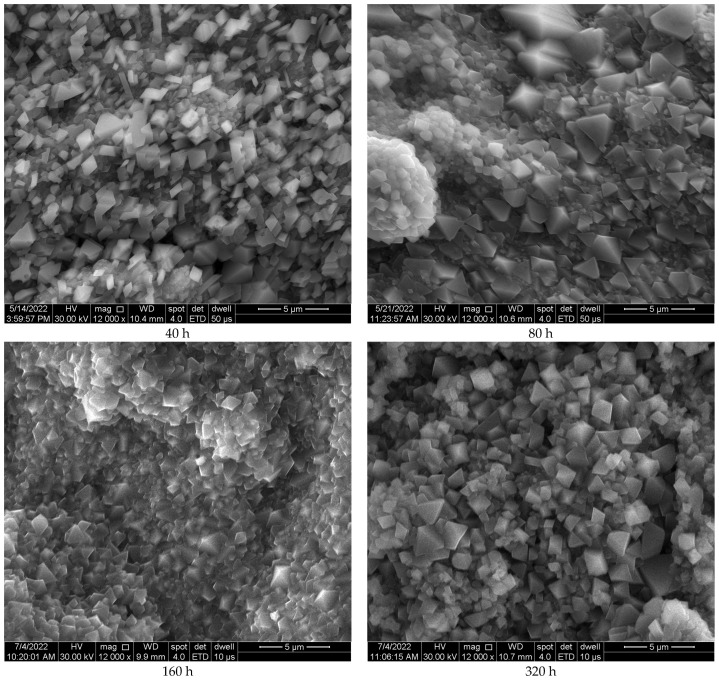
Representative SEM images presenting the morphology evolution of the oxide scale over 320 h of isothermal oxidation of CoCrMo at 914 °C.

Regarding the morphology evolution of the oxide scale, the images from Figure 3 reveal a rough surface oxide scale with rhombohedral morphology formed on the specimens’ surface. No significant morphological changes were observed in the oxide scale over the repeated exposure at 914 °C.

**Figure 4:**
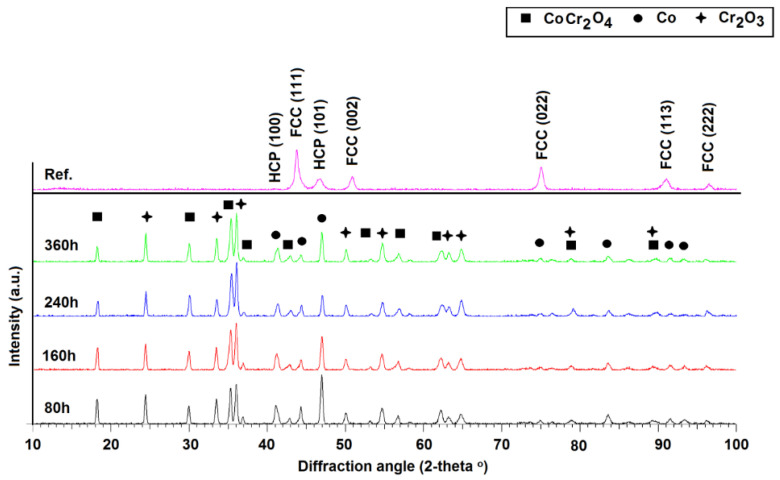
Resulting XRD patterns for the CoCrMo reference and CoCrMo specimens exposed at 914 °C for various durations.

Figure 4 presents the XRD patterns resulted for the CoCrMo reference and the CoCrMo oxidized specimens for various time durations.

Based on the patterns from Figure 4, it can be observed that the reference shows maximum diffraction peaks specific to FCC and HCP phases, the hexagonal phases being considered rich in Co. The FCC phase can be assumed to be the γ matrix, and the HCP phase could be small amounts of ε phase. Following high-temperature exposure for different time durations, an amorphous superficial layer was detected by the analysis of the material in grazing incidence diffraction (GID). Further, analyzing the material in Braag–Brentano geometry, the formation of some oxide phases was highlighted: respectively, a cubic spinel phase belonging to the space group Fd-3m (227) of CoCr_2_O_4_ (a = 8.41Å) and a rhombohedral phase with hexagonal axes belonging to the space group R-3c (167) of Cr_2_O_3_ (a = 4.98Å, c = 13.59Å). Additionally, there was also a hexagonal phase containing Co belonging to the space group P63/mmc (194) with the theoretical parameters of the elementary cell a = 2.53Å, c = 4.08Å, which is believed to be ε phase. The oxidative process at 914 °C promotes the martensitic transformation of γ to ε, as shown by the reduction in the FCC peaks along with the increase in the HCP peak intensity.

From the semi-quantitative Rietveld analysis, using the ICSD database (FIZ Karlsruhe) and the Topas analysis software (Bruker, Bremen, Germany), the evolution of the crystalline phases related to the duration of the exposure was highlighted and is presented in Table 2.

The XRD analysis revealed that the thermodynamically stable and protective Cr_2_O_3_ forms, and its amount increases over time. Besides Cr_2_O_3_, the spinel CoCr_2_O_4_ was identified as it forms due to the high content of Co, and the permanence at 914 °C favors the oxidation of Co. It can be observed from the values presented in Table 2 that the presence of Co and Cr in the outer material surface promotes the continuous formation of CoCr_2_O_4_, whose proportion increases over time, as the Cr_2_O_3_ proportion is maintained almost constant over time. The Cr_2_O_3_ scale can act as a barrier to further oxidation, protecting the base material against the oxygen diffusion; it is known to be denser than the CoCr_2_O_4,_ which is more porous, less protective, and susceptible to spallation. Furthermore, the thermal cycling at 914 °C promotes the martensitic transformation during the first cycles, then it decreases during the following stages of oxidation cycles.

**Figure 5:**
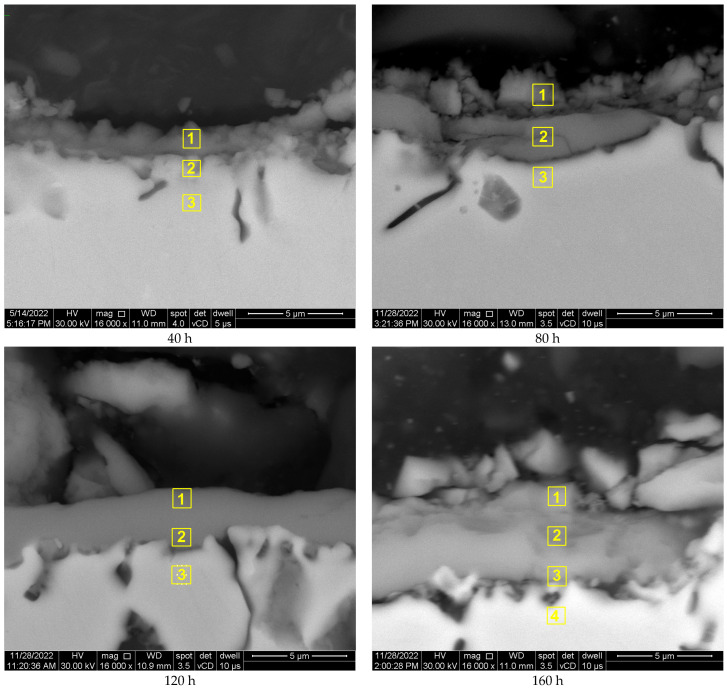

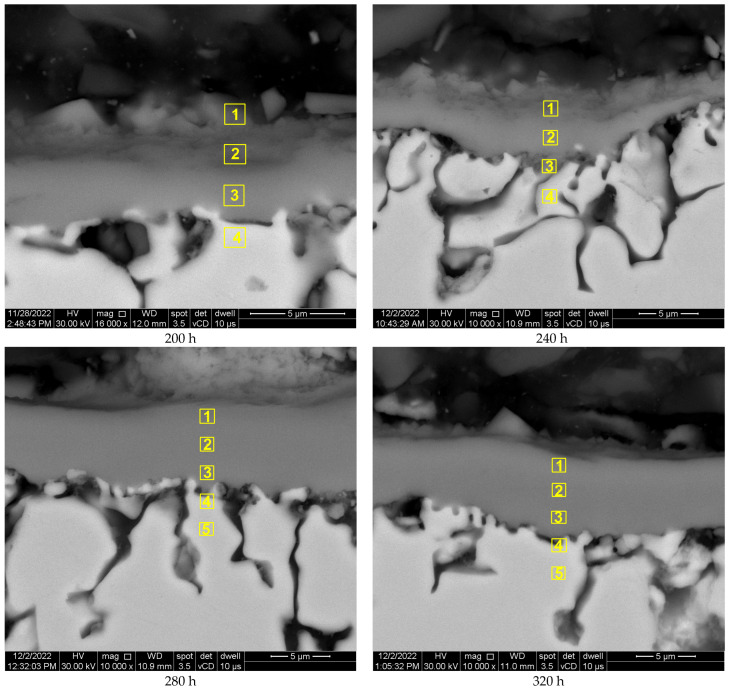
The micro-areas where the SEM-EDS analysis was performed (marked in yellow squares).

The chemical composition was determined in various micro-areas by SEM-EDS, as can be observed in the images from Figure 5. The analysis started by performing the measurements in one micro-area in the oxide scale region, but as the oxide scale thickness increases, the analysis was performed in more micro-regions. The chemical distribution analysis was analyzed only for three chemical elements of interest, O, Cr, and Co, as they form the oxide scale. The chemical distribution of the elements was presented for three representative areas, namely near the top surface of the oxide scale, at the base of oxide scale, and in the substrate (under the oxide scale).

**Figure 6:**
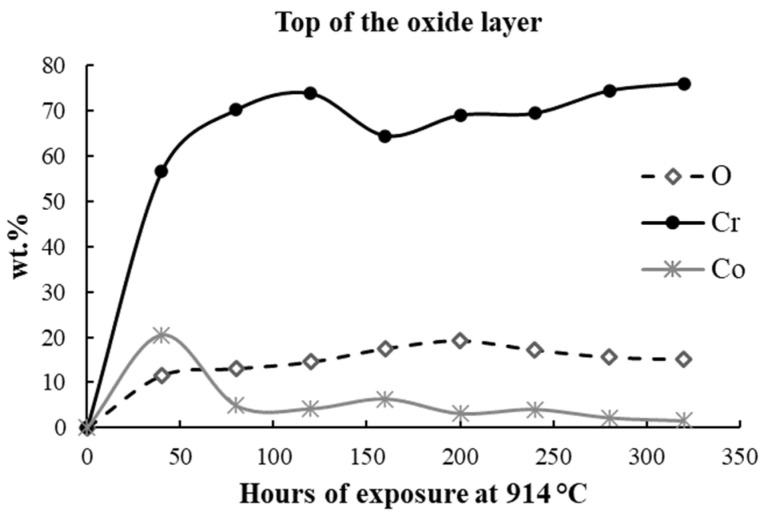
The evolution of O, Cr, and Co wt.% content in the top surface of the oxide scale.

**Figure 7:**
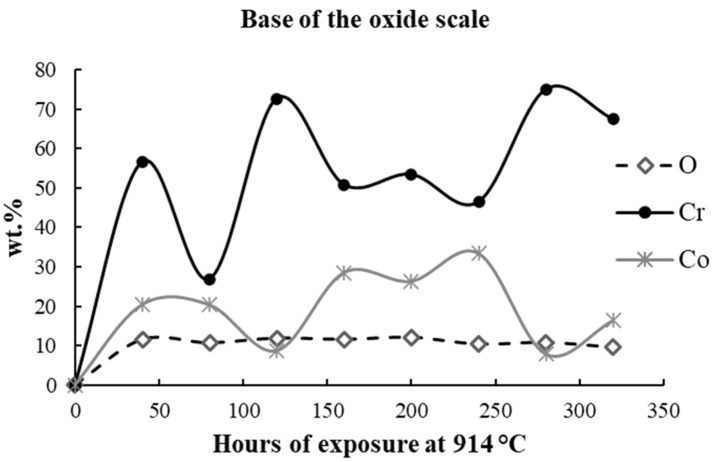
The evolution of O, Cr, and Co content in the base of the oxide scale.

**Figure 8:**
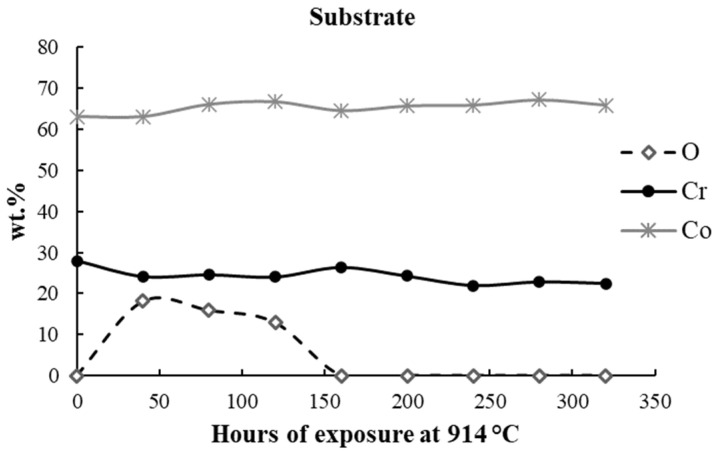
The evolution of O, Cr, and Co content in the substrate (the base material).

The elemental distribution is presented in weight percents (wt.%) in Table 3, Table 4 and Table 5 and Figure 6, Figure 7 and Figure 8.

The protective behavior can be demonstrated by the chemical analysis performed on the base material (substrate), as can be observed in Table 5 and Figure 8.

The evolution of the three elements of interest, O, Cr, and Co, over the 320 h exposure at 914 °C (Figure 6) showed an increase in Cr and O content over all the oxidation periods, a sign of the continuous formation of the Cr_2_O_3_, as was identified by XRD. The Co content increase was significant for the first 40 h of exposure, a sign of the formation of CoCr_2_O_4_ spinel, and further, the Co content is slightly declining during the following exposure cycles. 

Table 4 shows the content of O, Cr, and Co at the base of the oxide scale. In this region, a fluctuation in the Cr and Co content was found (mainly caused by the limitation of the measuring technique), but an increase in O was registered during the first 40 h of exposure, a sign of the active oxidative process. Further, a constant O content was maintained as the oxide scale was continuously formed.

Based on the values recorded for the O content, it can be observed that during the first oxidative cycles, the O is absorbed from the atmosphere by the superficial material’s layers, and further, this content is used along with Cr and Co to produce the oxide scale consisting in Cr_2_O_3_ and CoCr_2_O_4_. Since the 160 h of oxidation, the protective Cr_2_O_3_ does not allow the further penetration of O. 

**Figure 9:**
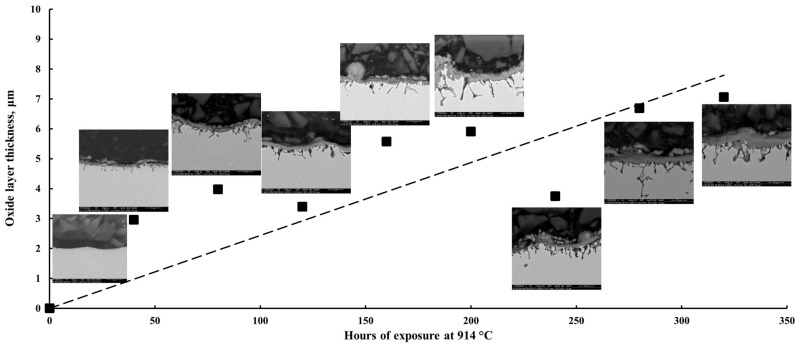
Oxide scale thickness as a function of time (SEM images with the oxide layer were made at a 5000× magnification).

Figure 9 shows the evolution of the oxide scale thickness. From this image, a linear increasing trend can be observed in the oxide scale thickness; it increases until 240 h of exposure at 914 °C, where spallation of the oxide scale was observed by SEM analysis and thickness measurements. Subsequently, the thickness of the oxide scale increases as the oxidation process continues, the high content of Cr and Co promoting the oxidative process.

**Figure 10:**
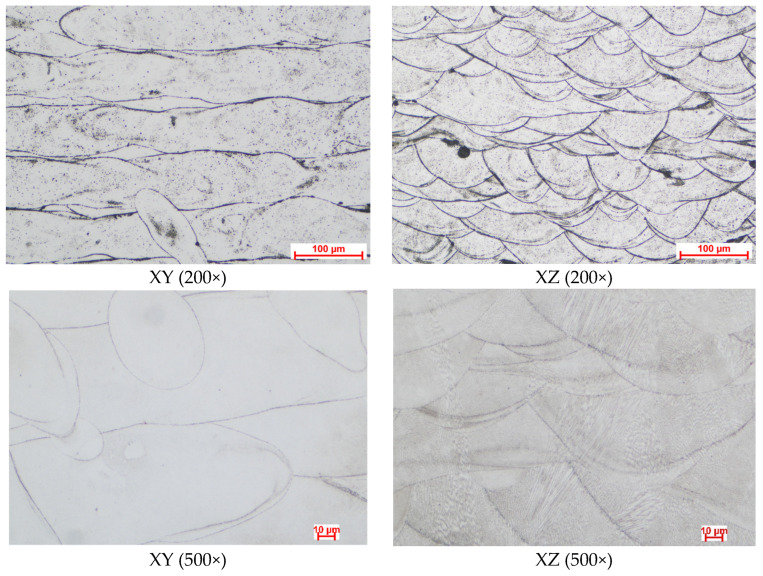
Representative optical microscopy images revealing the microstructural features of CoCrMo alloy in the as-printed state (magnification 200×, respective 500×).

By optical microscopy, the microstructural features of the material in the initial state and after multiple isothermal oxidation cycles at 914 °C were assessed. The typical microstructure of SLM manufactured materials was observed for the reference specimen (as-printed state), as can be observed in Figure 10. The melt pool tracks and melt pool boundaries were well visible in the XY plane, and melt pool overlapping was also observed, while in the XZ plane, the typical “fish scale” morphology was recorded. At higher magnification, fine cellular structures can be observed in the XY plane, while in the XZ plane, the epitaxial growth of grains was recorded. Columnar grains that extend through multiple layers following the heat flow direction, almost parallel to the building direction, were observed. As was determined by XRD, in the as-printed state, the material consists of the γ (FCC) and small traces of ε phase (HCP).

**Figure 11:**
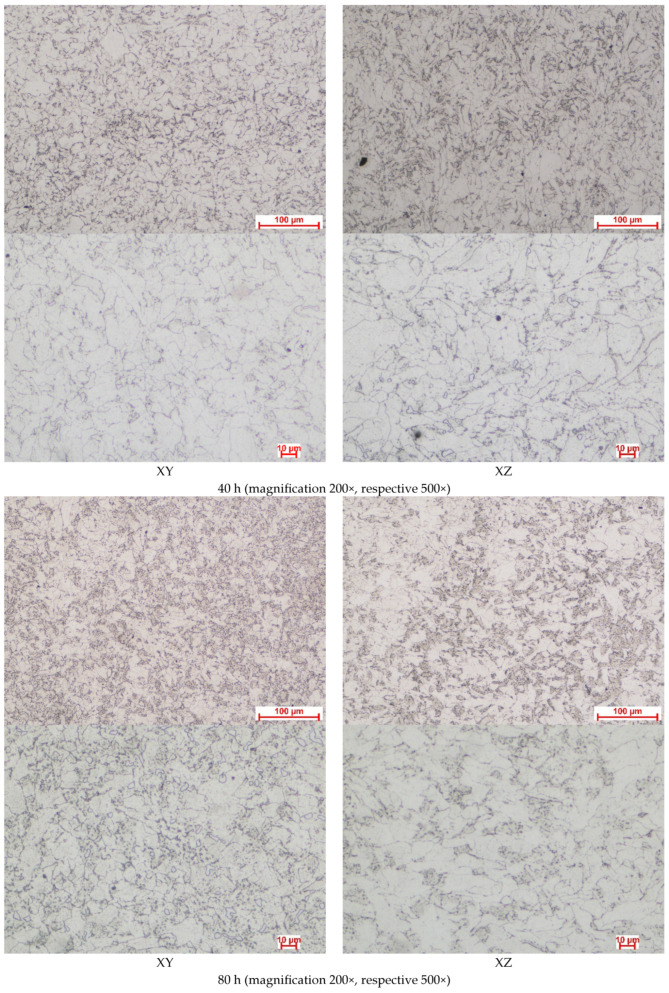

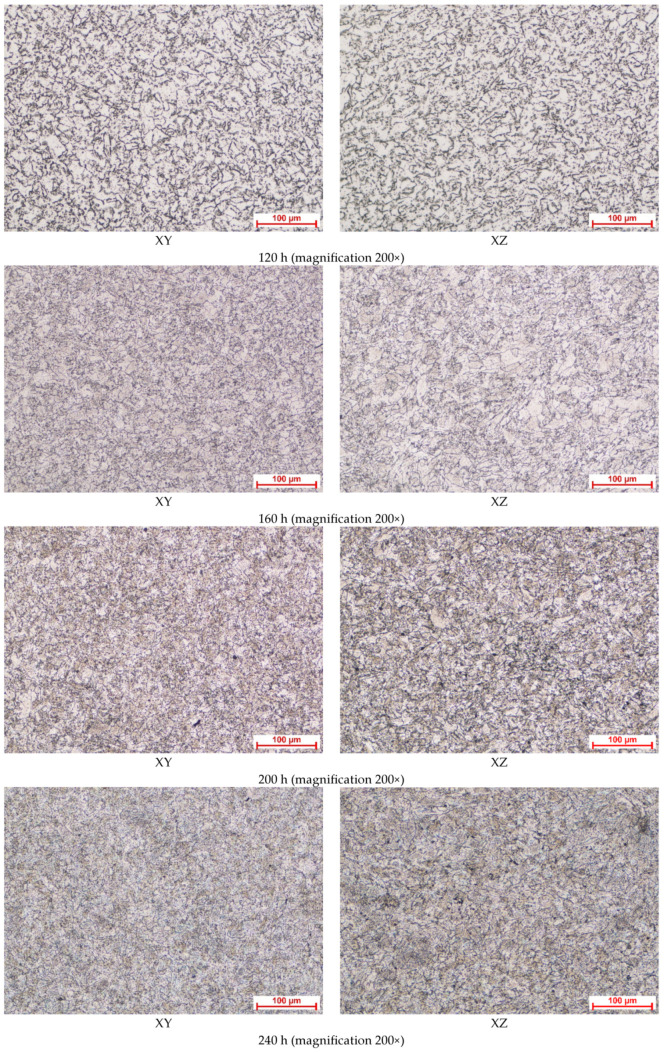

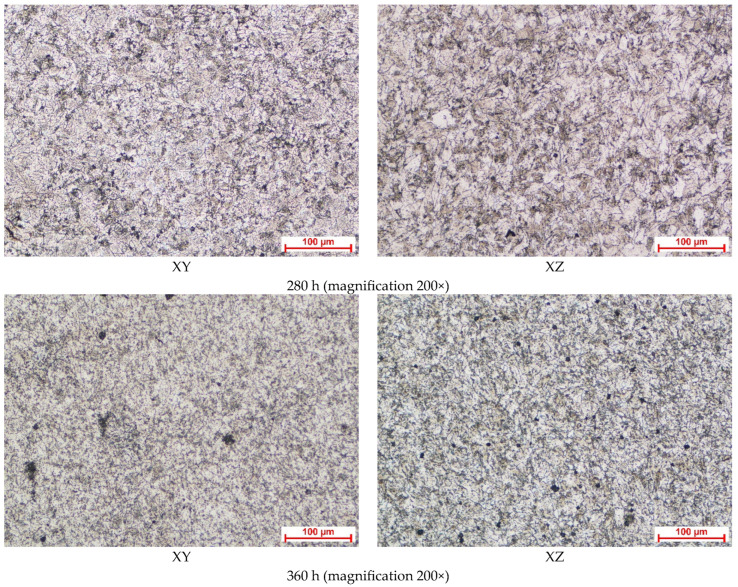
Representative optical microscopy images revealing the microstructural features of CoCrMo alloy after the isothermal oxidation cycles.

The complete recrystallization of the material can be observed after the high-temperature exposure. The evolution of the microstructure over all 320 h isothermal oxidation at 914 °C can be observed in the images from Figure 11.

It is believed, based on the optic microscopy images, that the prolonged exposure at 914 °C promoted carbide precipitation (temperature being lower than the conventional temperature for homogenization heat treatment, even lower than the final stage of the high-temperature stress relief treatment presented in the material’s data sheet, namely 1150 °C [30]). The 914 °C is in the temperature range specific to the precipitation hardening window for Co alloys rich in Cr with the goal of carbide precipitation at the grain boundaries (M_23_C_6_, M_7_C_3_), to enhance the material’s hardness, strength, and wear resistance. In our case, the exposure at 914 °C promoted the development of equiaxed grains on all planes analyzed, the grain boundaries being visible. Comparing the XRD patterns, it can be observed that HCP peaks from the reference sample increase in intensity during the oxidative cycles, probably because of the martensitic transformation of part of the γ phase into ε phase, and a precipitation of Co rich carbides (M_23_C_6_, M_7_C_3_ where M is Co) is possible. Fine precipitates were observed in the images presented in Figure 11.

**Figure 12:**
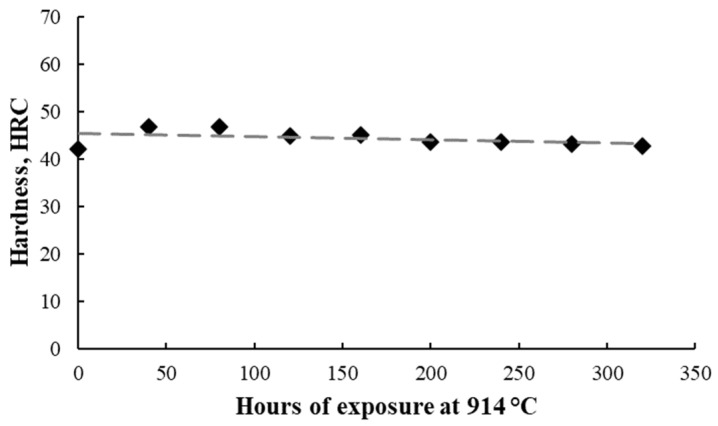
Hardness evolution of additive manufactured CoCrMo alloy.

The influence of the isothermal oxidation cycles on the material’s hardness was studied, with the material’s hardness evolution presented in the graph from Figure 12. In the as-printed state, a 42 HRC value was measured. The oxidation process ensured a slight increase in the hardness after the first 40 h of exposure, proving the higher proportion of ε martensite and carbide. Further, prolonged exposure at 914 °C for 320 h ensured a material hardness similar to the hardness of the material in the as-printed condition.

## 4. Discussion

The high-temperature cyclic isothermal oxidation behavior of an additive manufactured CoCrMo alloy with 0.14% C content was investigated progressively until a 320 h total duration of exposure at 914 °C was reached. The targeted application of the material is the manufacturing of various mandrels’ geometries for manufacturing metallic pipe fitting elbows. The heat treatment conditions were established based on the mandrel’s working conditions and its lifetime. A mass gain rate increase was registered during the first 40 h of exposure (reaching a value of 12.6 × 10^−6^ g·cm^−2^·h^−1^ after 40 h of exposure), with further observation of a decreasing behavior, reaching a value of 4.3 × 10^−6^ g ·cm^−2^·h^−1^ after 320 h of exposure.

Based on the mass gain analysis, the oxidation process kinetic curve for additively manufactured CoCrMo was determined, and it was observed that it follows a parabolic law in case of isothermal oxidation at 914 °C. The kp oxidation constant was calculated and, for all 320 h of exposure, was 16.7 × 10^−13^ g^2^·cm^−4^·s^−1^. The values recorded are consistent with other values presented by different authors. Buscail et al. [31] studied the isothermal oxidation of a conventional CoCr alloy at 800 °C, 900 °C, 1000 °C, and 1100 °C for 24 h and observed a parabolic behavior of the oxidized material. The kp oxidation constant identified for the CoCr alloy at 800 °C for 24 h was 1.68 × 10^−14^ g^2^·cm^−4^·s^−1^, 3.78 × 10^−13^ g^2^·cm^−4^·s^−1^ in case of 900 °C oxidation, 3.20 × 10^−12^ g^2^·cm^−4^·s^−1^ for 1000 °C oxidation, and 1.66 × 10^−11^ g^2^·cm^−4^·s^−1^ for 1100 °C oxidation. 

Wright and Wood [32] studied the oxidation of various conventional manufactured CoCr alloys in oxygen (pressure of 760 torr) at 1000 °C, and they obtained, for alloys with 30 wt.% Cr, kp values of 3.6 × 10^−9^ g^2^·cm^−4^·s^−1^, 5.4 × 10^−9^ g^2^·cm^−4^·s^−1^, 5 × 10^−10^ g^2^·cm^−4^·s^−1^, and 4.6 × 10^−10^ g^2^·cm^−4^·s^−1^, depending on the batch. Their kp values were higher than those obtained during the present study, but they oxidized the specimens in the oxygen environment. 

Based on the XRD analysis, it was concluded that the oxide scale formed during isothermal oxidation cycles consisted of a dense oxide Cr_2_O_3_ and the porous spinel CoCr_2_O_4_, these two being found also by Buscail et al. [31] along with other spinels and oxides in case of a CoCr alloy heat treated for 34 cycles at 900 °C, 1000 °C, and 1100 °C (heat treatment consisting in 20 h of exposure followed by 4 h of maintaining at ambient temperature after air quenching). The oxide scale identified by Wright and Wood [32] consisted in Cr_2_O_3_, CoCr_2_O_4_, Co_3_O_3_ and CoO for the Co alloy with 30 wt.% Cr.

During the present study, an oxide scale thickness in the range of 3–7 μm was registered, and it increases along with the increase in oxidation cycles. The only exception recorded was at 240 h of exposure when oxide scale spallation occurred. Even if the spallation occurred in some areas, it did not significantly affect the kinetic curve, and further, the oxidative process continued, increasing the oxide scale thickness. It is believed that the spallation occurred due to the porous nature of the CoCr_2_O_4_ spinel. Spallation of the oxide scale was found also by Buscail et al. [31], who observed that at 900 °C, the oxide scale presented a bad adherence related to the cobalt oxidation and the CoCr_2_O_4_ oxide formation. They obtained for specimens oxidized for 24 h at 800 °C a 1 μm thick scale, a 4 μm thick scale for the material oxidized at 900 °C, an 8 μm thick scale for the specimens oxidized at 1000 °C, and an 11 μm scale thickness resulted from the oxidation of the material at 1100 °C. Spallation of the oxide scale was also found by Wright and Wood [32].

During the present study, in the as-printed state, the CoCrMo alloy showed a typical microstructure for a SLM manufactured material including visible melt pool tracks and melt pool boundaries, and melt pools overlapping in the XY plane, while in the XZ plane, the typical “fish scale” morphology was identified. The epitaxial growth of grains was observed, and fine cellular structures were present in the XY plane, while in the XZ plane, columnar grains that extend through multiple layers following the heat flow direction, almost parallel to the building direction were observed. The XRD analysis determined that in the as-printed state, the material consists mainly in the γ phase with small traces of Co-rich ε phase. The complete recrystallization of the material occurred after the prolonged exposure at 914 °C; an increased ε phase proportion was registered at the 80 h of exposure at 914 °C that further decreases as the maintenance duration increases. Roudnicka et al. [33] studied the response of cast and additive manufactured CoCrMo alloy after heat treatment in the temperature range of 400–1000 °C and aging at 1200 °C for 2 h. They also observed epitaxial growth in the case of the SLM manufactured material. The heat treatment between 800 °C and 900 °C provided a recrystallized microstructure with fully HCP grains in the 3D-printed material, while precipitated particles of σ-phase, dendritic microstructure were preserved in the cast material in which lamellar HCP phase developed by isothermal martensitic transformation appeared in the original FCC Co matrix with extended carbidic domains.

In the present case, except for the dominant γ phase and small amounts of ε phase, the precipitation of fine of Co rich carbides (M_23_C_6_, M_7_C_3_ where M is Co) was considered possible, as can be observed in the microstructural images and in the XRD patterns.

Similar microstructural features were found by Zhang et al. [34] for SLM manufactured CoCrMo, during a study on its aging behavior. In the as-printed state of the material, they identified the predominant γ phase along with ε phase, the proportion changing after applying different heat treatments. Furthermore, the heat treatment promoted the precipitation of intermetallic compounds, such as Cr_2_N, MoC, and Cr_23_C_6_.

The conclusion is that during the oxidation of CoCrMo at 914 °C, fine carbide precipitate is supported not only by the microstructural images but also by the increase of 12% in the material’s hardness measured after the first stage of oxidation for 40 h of exposure at 914 °C. Roudnicka et al. [33] observed an increased hardness in the conventional material caused by the fine hard carbide precipitation combined with an increase in the HCP phase by isothermal transformation, a massive FCC to HCP transformation being the main cause of the hardness increase in the 3D-printed material.

The values recorded for the hardness of the additive manufactured CoCrMo with 0.14% C content were consistent with other registered values from the literature. Valerio-Vidal et al. [35] studied the influence of carbides and the microstructure of conventional manufactured CoCrMo alloys on their metallic dissolution resistance. For specimens containing 0.19 wt.% C, they obtained 410 HV_500g_ (which is around 42 HRC). SEM images presented by Valerio-Vidal et al. [35] for CoCrMo alloys heat-treated differently showed the presence of carbides, and the volume of carbides decreased with time and with the temperature used in heat treatments. A decrease in hardness was also found in the present study with the increase in high-temperature exposure.

Mas Ayu et al. [26] studied the thermal oxidation of CoCrMo alloys as a solution to improve the alloy’s corrosion resistance. Two different oxidation durations were used, 3 h and 6 h, at 850 °C. The Vickers hardness after corrosion testing was measured to assess the influence of thermal oxidation in reducing corrosion rate. They showed that the substrates that undergo thermal oxidation for 6 h performed better than those oxidized for 3 h, with the hardness values of 832.2 HV (around 65 HRC) and 588 HV (around 54.5 HRC), but for the untreated specimen, they obtained a 479.2 HV (approx. 48 HRC). Zhang et al. [34] measured a 425 HV microhardness (approx. 43 HRC) for additive manufactured CoCrMo in the as-printed state, reaching around 510 HV (approx. 50 HRC) after heat treatment. The increase in hardness was attributed to the martensitic transformation (from γ to ε) and precipitation. 

In the present study, the slight reduction in the material’s hardness after 80 h exposure at 914 °C can be caused by the reduction in the ε phase proportion but also by the defect formation in the material (increase in pores during prologued oxidation).

Analyzing the kinetic curve in relation with material’s hardness and microstructure, it can be said that small traces of ε phase along with carbides can ensure a slight increase in material hardness that can also improve the material’s oxidation resistance. The CoCrMo alloy exhibits a good oxidation resistance due to its compact microstructure that promotes the formation of a protective Cr_2_O_3_ oxide layer, hindering the diffusion of oxygen, affecting the oxidation kinetics by reducing the oxidation rate and ensuring a low kp value. A harder CoCrMo alloy with refined microstructure obtained during the first oxidation stages promoted the formation of robust oxide layers enhancing the oxidation resistance, but after multiple cycles, the stress in the material can also result in oxide cracking and spallation, which can affect its protective nature (a conclusion supported by the results recorded after oxidation stages for 240 h and 280 h of maintaining at 914 °C and by the oxide spallation observed experimentally).

As it is an alloy that operates under harsh conditions at high temperatures in air, it is capable of maintaining its structural integrity under the oxidative conditions during functioning.

## 5. Conclusions

An investigation into the high-temperature oxidation behavior of an additive manufactured CoCrMo alloy with 0.14 wt.% C was investigated as it was considered a possible candidate for mandrel manufacturing. The oxidation heat treatment was performed for 320 h of exposure at 914 °C. This experiment showed the following:

- The oxidative process follows a parabolic law, the process being fast during the first 40 h of maintaining at 914 °C, later slowing down.
- In the as-printed state, the material presented mainly the γ matrix and small traces of ε phase, while the oxidative process promoted the increase in the proportion of ε phase and the precipitation of carbides.
- The oxidation process during 320 h of exposure at 914 °C ensured the development of a scale consisting of a dense Cr_2_O_3_ layer and a porous layer of CoCr_2_O_4_ spinel.
- The oxide scale presented a rhombohedral morphology with no significant morphological changes over the oxidation thermal cycling at 914 °C.
- Oxide scale spallation was observed after 240 h of maintenance at 914 °C due to the porous nature of the CoCr_2_O_4_ spinel.
- In the as-printed state, a 42 HRC value was measured that increased by 12% after the first 40 h of exposure (proving the higher proportion of ε martensite and carbides precipitation). Further, the oxidation process ensured a reduction in the material hardness (similar to the hardness of the material in the as-printed condition) that could be a cause of the reduction in the ε phase proportion but also of the defect formation in the material (increase in pores during prologued oxidation).

During the present study, no mechanical loads were considered, and further studies targeting the mechanical response of the material under loading at high temperatures are worth considering, as well as future studies with mandrels’ manufacturing trials and the validation of experimental mandrels.

## Figures and Tables

**Table 1:** Chemical composition of additive manufactured CoCr MP1.

Chemical Composition	Weight Percents, wt.%							
	Co	Cr	Mo	Si	Mn	Fe	C	Ni
Technical datasheet [29]	60–65	26–30	5–7	Max. 1.0	Max. 1.0	Max. 0.75	Max. 0.16	Max. 0.1
Experimentally determined by SEM-EDS	63.00	28.00	5.50	0.86	0.90	0.70	0.14	0.10

**Table 2:** The evolution of the crystalline phases related to the duration of the exposure in case of 914 °C isothermal oxidation of additive manufactured CoCrMo alloy.

Exposure Time, h	CoCr_2_O_4_, %	Cr_2_O_3_, %	HCP Rich in Co, %
80	21.4	65.6	13.0
160	27.6	64.8	7.6
240	30.0	65.2	4.8
320	30.7	63.7	5.6

**Table 3:** The chemical analysis determined at the top surface of oxide scale.

Micro-Areas Where Eds Analysis Was Performed	Hours of Exposure at 914 °C	Weight Percent (wt.%)		
		O	Cr	Co
1	0	0	0	0
	40	11.5	56.6	20.4
	80	13.0	70.2	4.9
	120	14.5	73.8	4.2
	160	17.5	64.5	6.4
	200	19.2	69.0	3.2
	240	17.2	69.5	4.1
	280	15.6	74.4	2.2
	320	15.1	76.1	1.6

**Table 4:** The chemical analysis determined at the base of oxide scale.

Micro-Areas Where EDS Analysis Was Performed	Hours of Exposure at 914 °C	Weight Percent (wt.%)		
		O	Cr	Co
-	0	0	0	0
1	40	11.5	56.6	20.4
2	80	10.7	26.9	20.3
2	120	11.9	72.7	8.7
3	160	11.6	50.9	28.5
3	200	12.1	53.5	26.3
3	240	10.4	46.5	33.4
3	280	10.8	75.0	7.8
3	320	9.5	67.5	16.3

**Table 5:** The chemical analysis determined in the substrate.

Micro-Areas Where EDS Analysis Was Performed	Hours of Exposure at 914 °C	Weight Percent (wt.%)		
		O	Cr	Co
reference	0	0	28	63
3	40	18.2	24.2	63.0
3	80	16.0	24.6	66.1
3	120	13.0	24.0	66.7
4	160	0.0	26.4	64.5
4	200	0	24.3	65.6
4	240	0	22.0	65.8
5	280	0.0	22.9	67.1
5	320	0.0	22.5	65.9

## Data Availability

The data that support the findings of this study are available from the corresponding author, upon reasonable request.
